# Supplementary material for: Comparison of rhesus and cynomolgus macaques as an infection model for COVID-19
Source: Nat Commun. 2021 Feb 24;12:1260. doi: 10.1038/s41467-021-21389-9 (PMC7904795; doi:10.1038/s41467-021-21389-9)
Supplement: Supplementary file 1 — Supplementary Information [file 41467_2021_21389_MOESM1_ESM.pdf]

Supplementary Information

Comparison of Rhesus and Cynomolgus macaques as an infection model for COVID-19.

Viral RNA detected by RT-qPCR in nasal cavity, lung, trachea and tonsil.

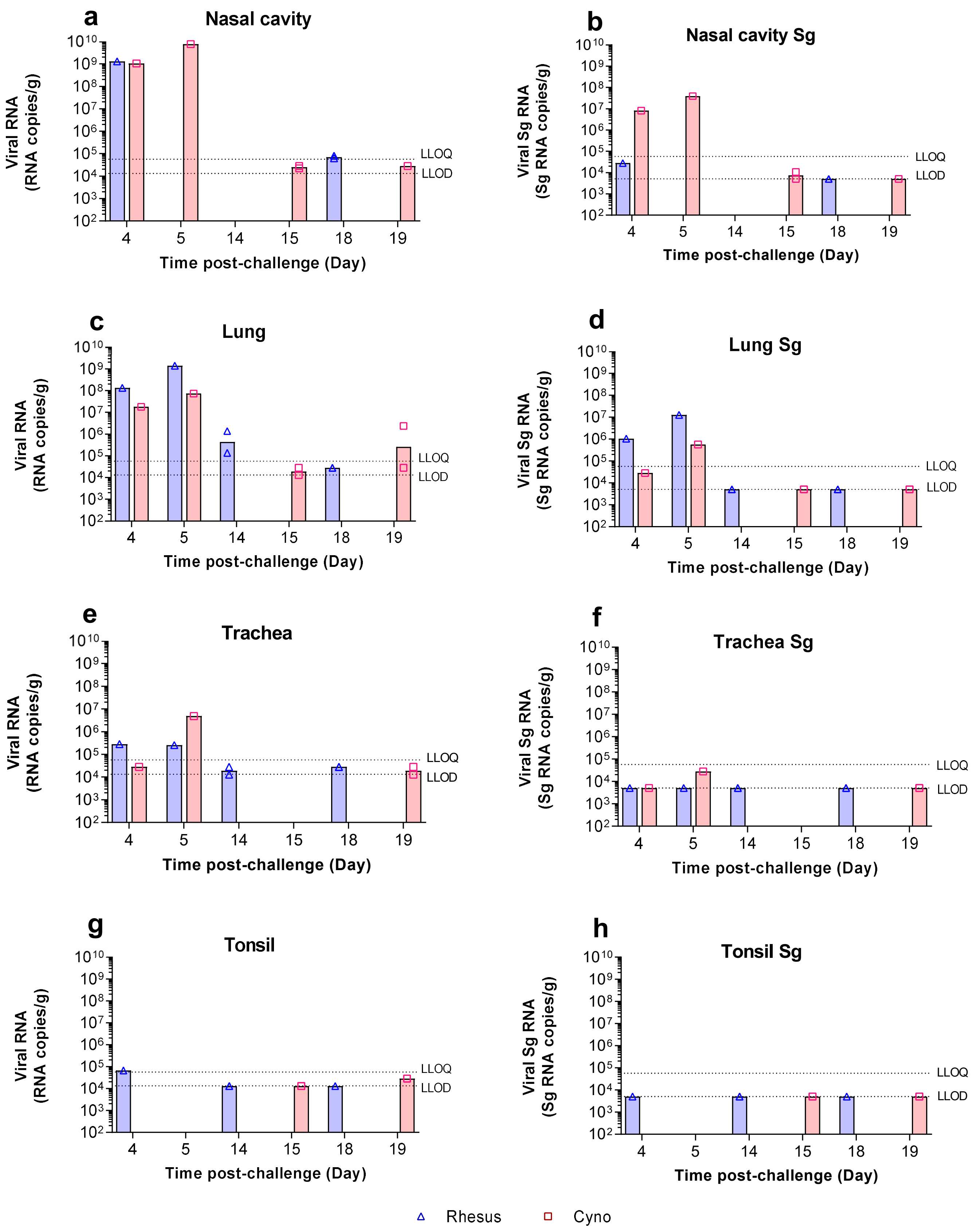

Supplementary Figure 1. Viral RNA detected by RT-qPCR in nasal cavity, lung, trachea and tonsil. Viral load presented as the geometric mean of RNA copies/ml or individual values, with individual data points overlaid in rhesus macaques (blue) and cynomolgus macaques (red) in (**a**) nasal cavity total RNA , (**b**) nasal cavity Sg RNA, (**c**) lung total RNA , (**d**) lung Sg RNA (**e**) trachea total RNA , (**f**) trachea Sg RNA, (**g**) tonsil total RNA , (**h**) tonsil Sg RNA. Numbers indicate days post challenge the NHP was euthanised. *n*= *1* macaques per group/species at 4 and 5dpc; *n*=2 at 14, 15, 18 and 19dpc. Bars show median values. Dashed lines highlight the LLOQ (lower limit of quantification, 5.71 x 10^4 copies/g for total RNA and subgenomic RNA) and LLOD (lower limit of detection, 1.31 x10^4 copies/g for total RNA, 5.14 x10^3 copies/g for subgenomic RNA). Positive samples detected below the LLOQ were assigned the value of 2.86 x 10^4 copies/g for total RNA and subgenomic RNA. Viral RNA was not detected in naïve animals. Samples for RT-qPCR and sgPCR were assayed in duplicate against a standard curve in triplicate.

Gross pathology

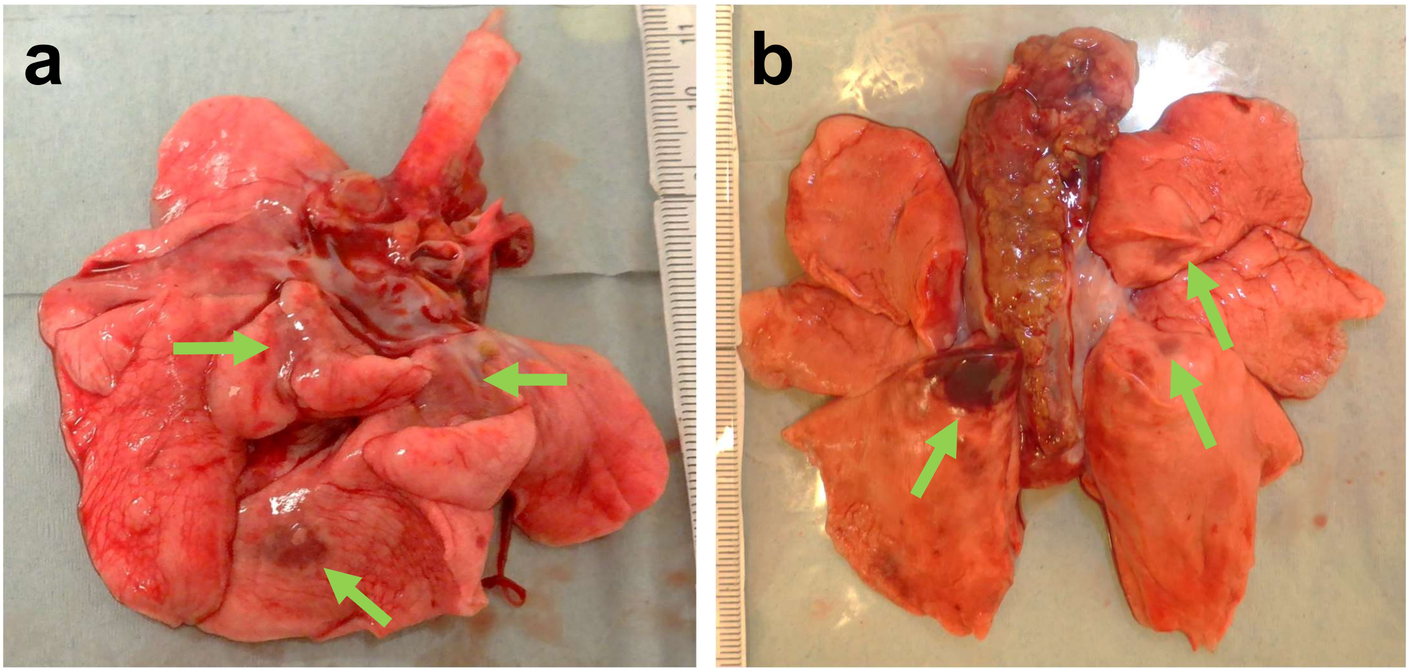

Supplementary Figure 2. Gross pathology in cynomolgus and rhesus macaques inoculated with SARS-CoV-2 culled at 4/5 dpc. (**a**) Ventral view of lung from a cynomolgus macaque showing multiple foci of moderate consolidation (arrows). (**b**) Dorsal view of lung from a rhesus macaque showing multiple foci of consolidation and congestion (arrows).

Consensus module – trait relationship between cynomolgus and rhesus macaques

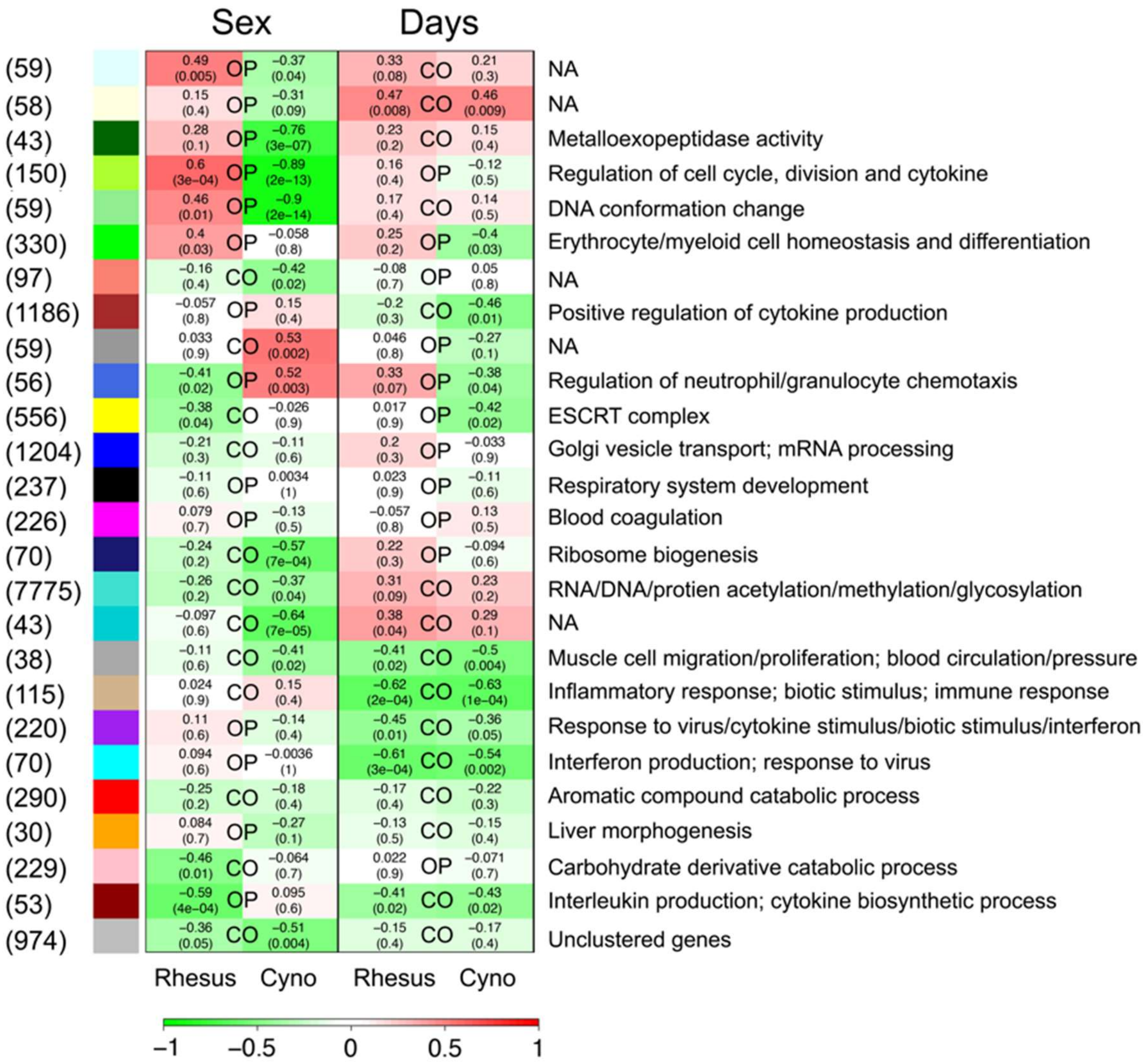

Supplementary Figure 3. Consensus module – trait relationship between cynomolgus and rhesus macaques. Clusters of genes of similar function and patterns of expression that were used to form consensus modules between cynomolgus and rhesus macaques. The numbers of genes that contribute to the consensus module are shown to the left and the description of that consensus module is shown to the right. (NA is that the module is of unknown function but is retained as gene annotation improves and the data can be re-analysed). The data is divided into two traits: sex of the animal and days post-infection. The upper number in each box is the strength of the relationship to the module. A value of 1 means the module is strongly positive (red) and associated with the trait and a value of -1 means the module is strongly negative (green) and not associated with the trait. The lower number in each box in parenthesis is the *P*-value for the relationship (positive or negative) for the module. A *P* value <0.05 is significant (one-sided).

Negative and positive controls for in situ hybridisation techniques to detect virus RNA and IL-6 mRNA.

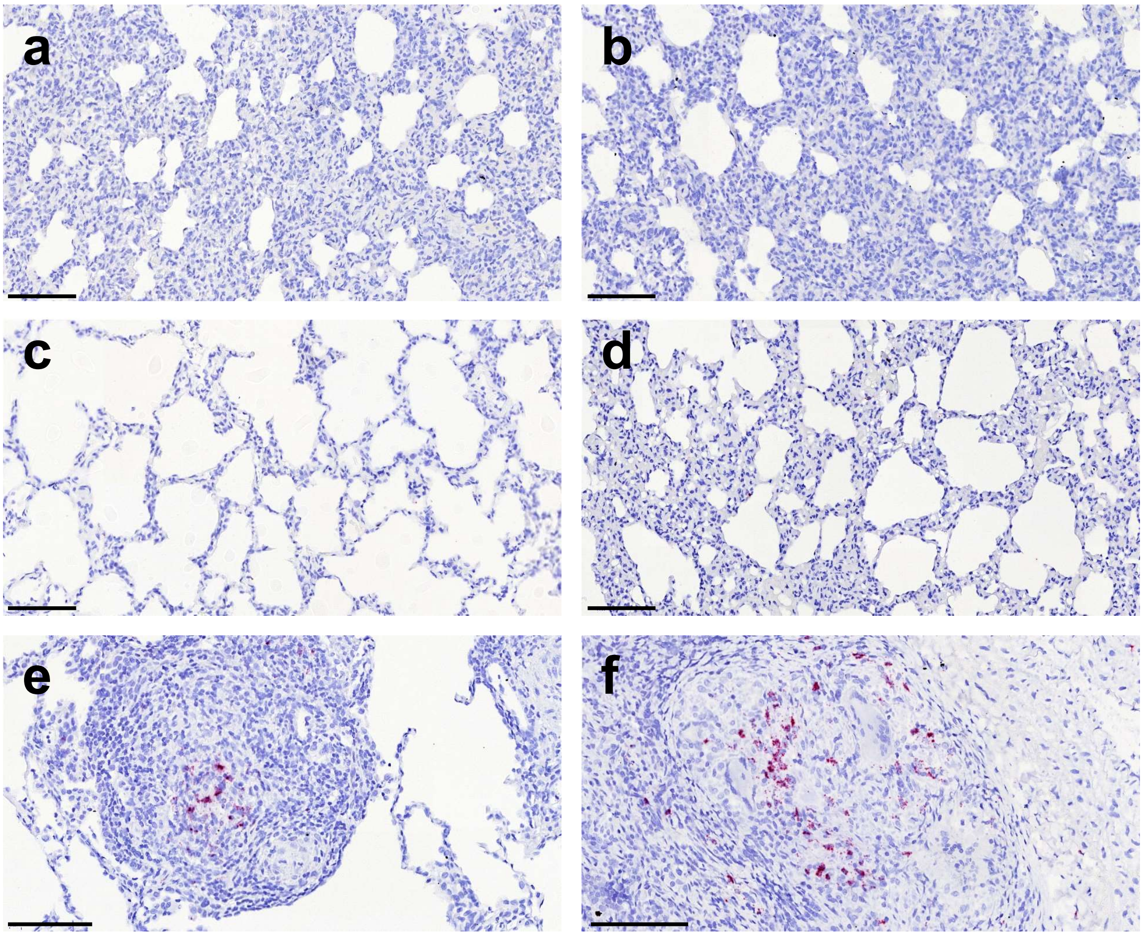

Supplementary Figure 4. Negative and positive controls for in situ hybridisation techniques to detect virus RNA and IL-6 mRNA. Negative control slides for ISH runs showing no staining in a rhesus macaque (**a**) and cynomolgus macaque (**b**) culled at 4 dpc. An irrelevant non-specific probe was used replacing the specific SARS-CoV-2 S-gene or IL-6 probes. Negative control naïve rhesus (**c**) or cynomolgus macaques (**d**) in ISH runs to detect SARS-CoV-2 S-gene. No stating was observed in naïve animals (archived samples from animals culled for non-infectious welfare reasons). Positive control slides to detect IL-6 mRNA using specific probes in rhesus (**e**) or cynomolgus (**f**) macaques from a *Mycobacterium tuberculosis* infection experiment. IL-6 mRNA (red staining) is shown within granulomas. Bars = 100µm

Flow cytometry gating strategy – intracellular staining

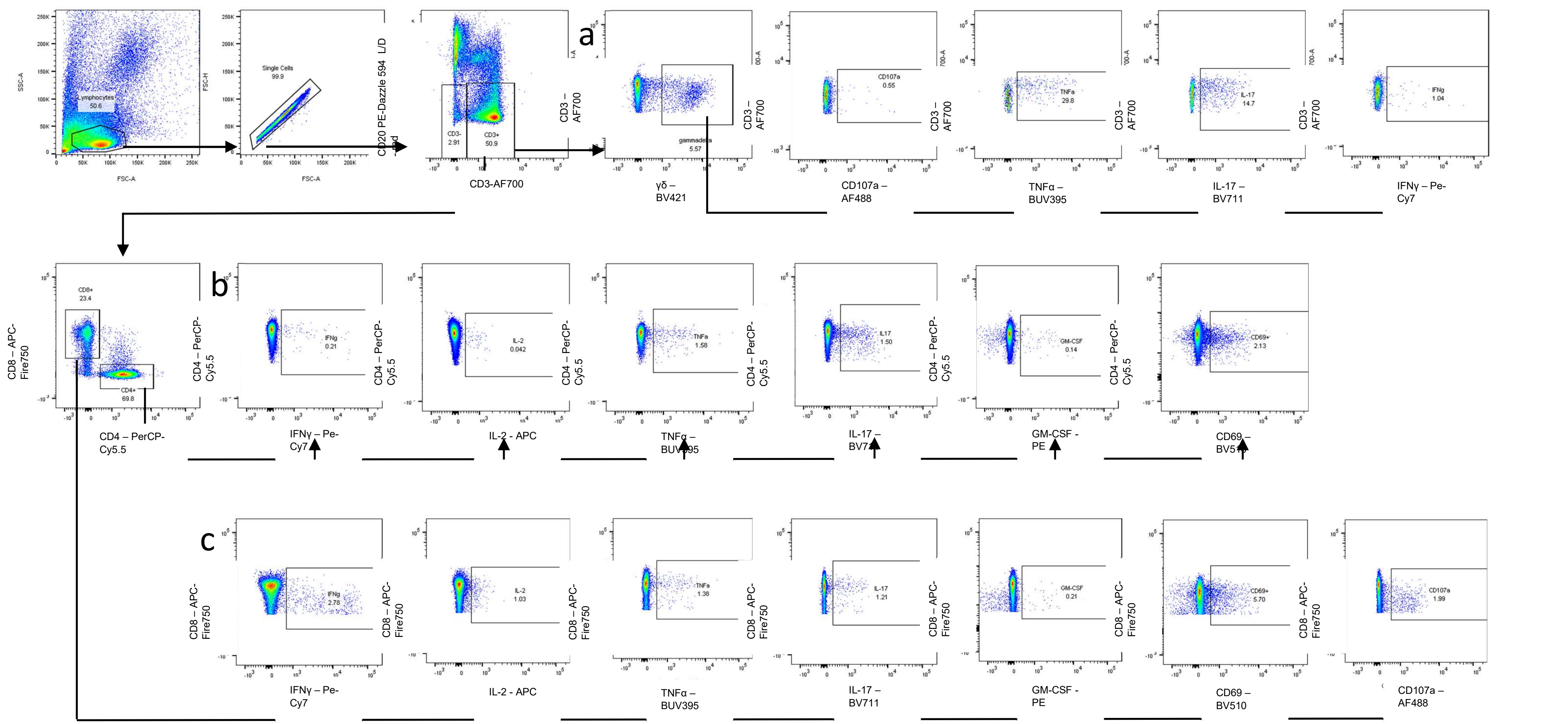

Supplementary Figure 5: Intracellular cytokine staining of PBMC and lung MNCs for γδ T-cells, CD4+ and CD8+ T-cells secreting IFNγ , TNFα, IL-2, IL-17, GM-CSF, CD107a or expressing CD69 used to obtain the data presented in Figure 9G, H, K, L, M, N.

Flow cytometry gating strategy – PBMC and lung MNC immunophenotyping

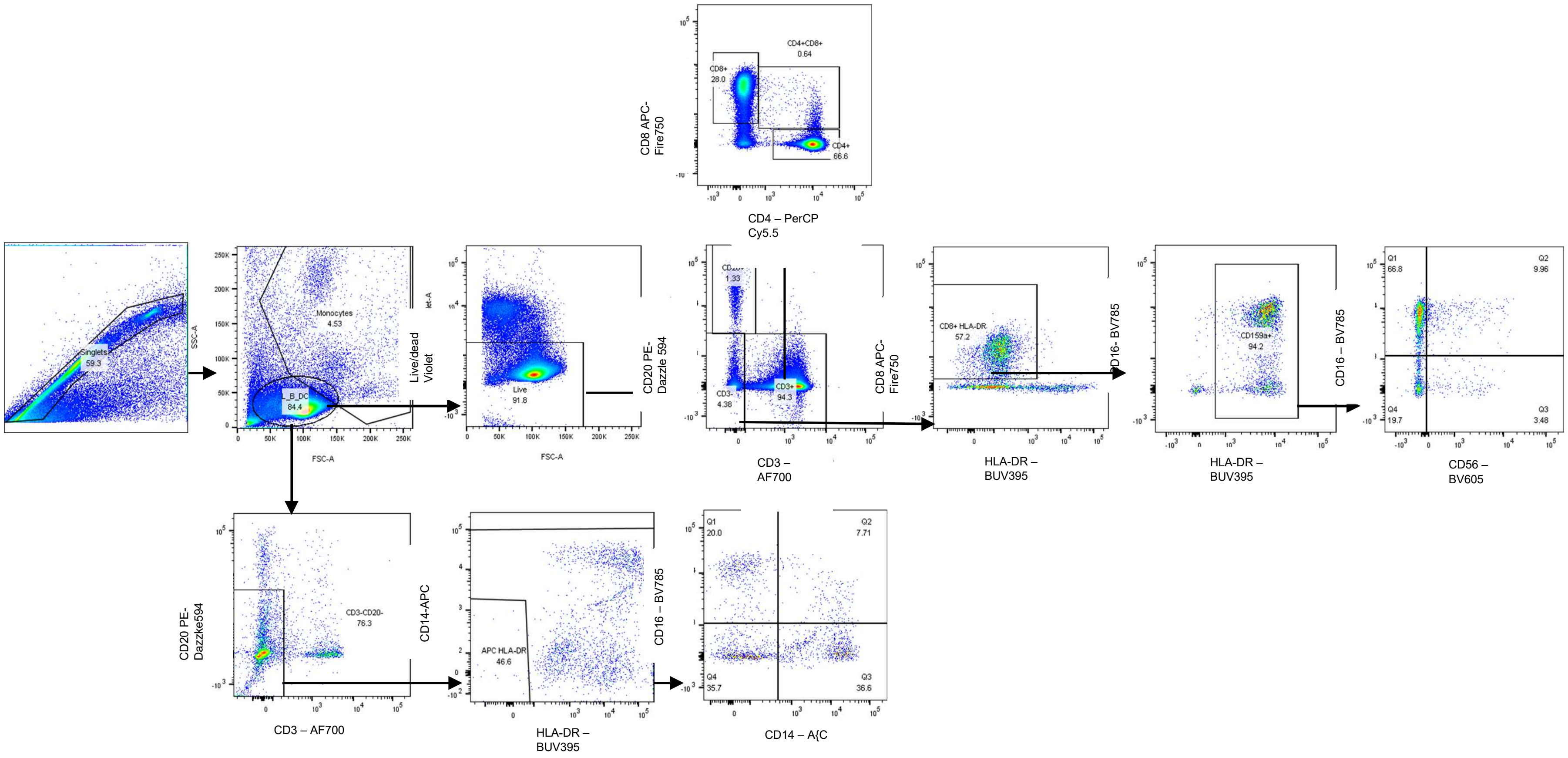

Supplementary Figure 6: Immunophenotyping of PBMC and lung MNCs for CD4+ and CD8+ T-cells, NK cells, monocytes, DCs used to obtain the data presented in Figure 9F, I and J.

Supplementary Table 1. Sequences of the primers and probes. Name and sequence of primers and probes utilised in RT-qPCR and sub-genomic PCR methods.

Name	Sequence
2019-nCoV_N1-forward	5' GACCCCAAAATCAGCGAAAT 3'
2019-nCoV_N1-reverse	5' TCTGGTTACTGCCAGTTGAATCTG 3'
2019-nCoV_N1-probe	5' FAM-ACCCCGCATTACGTTTGGTGGACC-BHQ1 3'
2019-nCoV_sgE-forward	5' CGATCTCTTGTAGATCTGTTCTC 3'
2019-nCoV_ sgE-reverse	5' ATATTGCAGCAGTACGCACACA 3'
2019-nCoV_ sgE-probe	5' FAM-ACACTAGCCATCCTTACTGCGCTTCG- BHQ1 3'

Supplementary Table 2: SARS-CoV-2 spike protein peptide library

Peptide No	NTerm	Sequence	CTerm	Pool	Peptides covered	Regions
1	H-	MFVFLVLLPLVSSQC	-OH	pool 1	1-139	Signal peptide
2	H-	LVLLPLVSSQCVNLT	-OH			S1 (14-685)
3	H-	PLVSSQCVNLTTRTQ	-OH			
4	H-	SQCVNLTTRTQLPPA	-OH			
5	H-	NLTTRTQLPPAYTNS	-OH			
6	H-	RTQLPPAYTNSFTRG	-OH			
7	H-	PPAYTNSFTRGVYYP	-OH			
8	H-	TNSFTRGVYYPDKVF	-OH			
9	H-	TRGVYYPDKVFRSSV	-OH			
10	H-	YYPDKVFRSSVLHST	-OH			
11	H-	KVFRSSVLHSTQDLF	-OH			
12	H-	SSVLHSTQDLFLPFF	-OH			
13	H-	HSTQDLFLPFFSNVT	-OH			
14	H-	DLFLPFFSNVTWFHA	-OH			
15	H-	PFFSNVTWFHAIHVS	-OH			
16	H-	NVTWFHAIHVSGTNG	-OH			
17	H-	FHAIHVSGTNGTKRF	-OH			
18	H-	HVSGTNGTKRFDNPV	-OH			
19	H-	TNGTKRFDNPVLPFN	-OH			
20	H-	KRFDNPVLPFNDGVY	-OH			
21	H-	NPVLPFNDGVYFAST	-OH			
22	H-	PFNDGVYFASTEKSN	-OH			
23	H-	GVYFASTEKSNIIRG	-OH			
24	H-	ASTEKSNIIRGWIFG	-OH			
25	H-	KSNIIRGWIFGTTLD	-OH			
26	H-	IRGWIFGTTLDSKTQ	-OH			
27	H-	IFGTTLDSKTQSLLI	-OH			
28	H-	TLDSKTQSLLIVNNA	-OH			
29	H-	KTQSLLIVNNATNVV	-OH			
30	H-	LLIVNNATNVVIKVC	-OH			
31	H-	NNATNVVIKVCEFQF	-OH			
32	H-	NVVIKVCEFQFCNDP	-OH			
33	H-	KVCEFQFCNDPFLGV	-OH	Pool 2	129-267	
34	H-	FQFCNDPFLGVYYHK	-OH			
35	H-	NDPFLGVYYHKNNKS	-OH			
36	H-	LGVYYHKNNKSWMES	-OH			
37	H-	YHKNNKSWMESEFRV	-OH			
38	H-	NKSWMESEFRVYSSA	-OH			
39	H-	MESEFRVYSSANNCT	-OH			
40	H-	FRVYSSANNCTFEYV	-OH			
41	H-	SSANNCTFEYVSQPF	-OH			
42	H-	NCTFEYVSQPFLMDL	-OH			

43	H-	EYVSQPFLMDLEGKQ	-OH			
44	H-	QPFLMDLEGKQGNFK	-OH			
45	H-	MDLEGKQGNFKNLRE	-OH			
46	H-	GKQGNFKNLREFVFK	-OH			
47	H-	NFKNLREFVFKNIDG	-OH			
48	H-	LREFVFKNIDGYFKI	-OH			
49	H-	VFKNIDGYFKIYSKH	-OH			
50	H-	IDGYFKIYSKHTPIN	-OH			
51	H-	FKIYSKHTPINLVRD	-OH			
52	H-	SKHTPINLVRDLPQG	-OH			
53	H-	PINLVRDLPQGFSAL	-OH			
54	H-	VRDLPQGFSALEPLV	-OH			
55	H-	PQGFSALEPLVDLPI	-OH			
56	H-	SALEPLVDLPIGINI	-OH			
57	H-	PLVDLPIGINITRFQ	-OH			
58	H-	LPIGINITRFQTLLA	-OH			
59	H-	INITRFQTLLALHRS	-OH			
60	H-	RFQTLLALHRSYLTP	-OH			
61	H-	LLALHRSYLTPGDSS	-OH			
62	H-	HRSYLTPGDSSSGWT	-OH			
63	H-	LTPGDSSSGWTAGAA	-OH			
64	H-	DSSSGWTAGAAAYYV	-OH			
65	H-	GWTAGAAAYYVGYLQ	-OH			
66	H-	GAAAYYVGYLQPRTF	-OH			
67	H-	YYVGYLQPRTFLLKY	-OH			
68	H-	YLQPRTFLLKYNENG	-OH			
69	H-	RTFLLKYNENGTITD	-OH			
70	H-	LKYNENGTITDAVDC	-OH			
71	H-	ENGTITDAVDCALDP	-OH			
72	H-	ITDAVDCALDPLSET	-OH			
73	H-	VDCALDPLSETKCTL	-OH			
74	H-	LDPLSETKCTLKSFT	-OH			
75	H-	SETKCTLKSFTVEKG	-OH			
76	H-	CTLKSFTVEKGIYQT	-OH			
77	H-	SFTVEKGIYQTSNFR	-OH	**Pool 3**	**257-395**	
78	H-	EKGIYQTSNFRVQPT	-OH			
79	H-	YQTSNFRVQPTESIV	-OH			
80	H-	NFRVQPTESIVRFPN	-OH			
81	H-	QPTESIVRFPNITNL	-OH			
82	H-	SIVRFPNITNLCPFG	-OH			
83	H-	FPNITNLCPFGEVFN	-OH			
84	H-	TNLCPFGEVFNATRF	-OH			**RBD (319-451)**
85	H-	PFGEVFNATRFASVY	-OH			
86	H-	VFNATRFASVYAWNR	-OH			
87	H-	TRFASVYAWNRKRIS	-OH			
88	H-	SVYAWNRKRISNCVA	-OH			
89	H-	WNRKRISNCVADYSV	-OH			

90	H-	RISNCVADYSVLYNS	-OH			
91	H-	CVADYSVLYNSASFS	-OH			
92	H-	YSVLYNSASFSTFKC	-OH			
93	H-	YNSASFSTFKCYGVS	-OH			
94	H-	SFSTFKCYGVSPTKL	-OH			
95	H-	FKCYGVSPTKLNDLC	-OH			
96	H-	GVSPTKLNDLCFTNV	-OH			
97	H-	TKLNDLCFTNVYADS	-OH	**Pool 4**	**385-523**	
98	H-	DLCFTNVYADSFVIR	-OH			
99	H-	TNVYADSFVIRGDEV	-OH			
100	H-	ADSFVIRGDEVRQIA	-OH			
101	H-	VIRGDEVRQIAPGQT	-OH			
102	H-	DEVRQIAPGQTGKIA	-OH			
103	H-	QIAPGQTGKIADYNY	-OH			
104	H-	GQTGKIADYNYKLPD	-OH			
105	H-	KIADYNYKLPDDFTG	-OH			
106	H-	YNYKLPDDFTGCVIA	-OH			
107	H-	LPDDFTGCVIAWNSN	-OH			
108	H-	FTGCVIAWNSNNLDS	-OH			
109	H-	VIAWNSNNLDSKVGG	-OH			
110	H-	NSNNLDSKVGGNYNY	-OH			
111	H-	LDSKVGGNYNYLYRL	-OH			
112	H-	VGGNYNYLYRLFRKS	-OH			
113	H-	YNYLYRLFRKSNLKP	-OH			
114	H-	YRLFRKSNLKPFERD	-OH			
115	H-	RKSNLKPFERDISTE	-OH			
116	H-	LKPFERDISTEIYQA	-OH			
117	H-	ERDISTEIYQAGSTP	-OH			
118	H-	STEIYQAGSTPCNGV	-OH			
119	H-	YQAGSTPCNGVEGFN	-OH			
120	H-	STPCNGVEGFNCYFP	-OH			
121	H-	NGVEGFNCYFPLQSY	-OH			
122	H-	GFNCYFPLQSYGFQP	-OH			
123	H-	YFPLQSYGFQPTNGV	-OH			
124	H-	QSYGFQPTNGVGYQP	-OH			
125	H-	FQPTNGVGYQPYRVV	-OH			
126	H-	NGVGYQPYRVVVLSF	-OH			
127	H-	YQPYRVVVLSFELLH	-OH			
128	H-	RVVVLSFELLHAPAT	-OH			
129	H-	LSFELLHAPATVCGP	-OH	**Pool 5**	**515-651**	
130	H-	LLHAPATVCGPKKST	-OH			
131	H-	PATVCGPKKSTNLVK	-OH			
132	H-	CGPKKSTNLVKNKCV	-OH			
133	H-	KSTNLVKNKCVNFNF	-OH			**S1 (14-685)**
134	H-	LVKNKCVNFNFNGLT	-OH			
135	H-	KCVNFNFNGLTGTGV	-OH			
136	H-	FNFNGLTGTGVLTES	-OH			
137	H-	GLTGTGVLTESNKKF	-OH			

138	H-	TGVLTESNKKFLPFQ	-OH			
139	H-	TESNKKFLPFQQFGR	-OH			
140	H-	KKFLPFQQFGRDIAD	-OH			
141	H-	PFQQFGRDIADTTDA	-OH			
142	H-	FGRDIADTTDAVRDP	-OH			
143	H-	IADTTDAVRDPQTLE	-OH			
144	H-	TDAVRDPQTLEILDI	-OH			
145	H-	RDPQTLEILDITPCS	-OH			
146	H-	TLEILDITPCSFGGV	-OH			
147	H-	LDITPCSFGGVSVIT	-OH			
148	H-	PCSFGGVSVITPGTN	-OH			
149	H-	GGVSVITPGTNTSNQ	-OH			
150	H-	VITPGTNTSNQVAVL	-OH			
151	H-	GTNTSNQVAVLYQDV	-OH			
152	H-	SNQVAVLYQDVNCTE	-OH			
153	H-	AVLYQDVNCTEVPVA	-OH			
154	H-	QDVNCTEVPVAIHAD	-OH			
155	H-	CTEVPVAIHADQLTP	-OH			
156	H-	PVAIHADQLTPTWRV	-OH			
157	H-	HADQLTPTWRVYSTG	-OH			
158	H-	LTPTWRVYSTGSNVF	-OH			
159	H-	WRVYSTGSNVFQTRA	-OH			
160	H-	STGSNVFQTRAGCLI	-OH			
161	H-	NVFQTRAGCLIGAEH	-OH	**Pool 6**	**643-779**	
162	H-	TRAGCLIGAEHVNNS	-OH			
163	H-	CLIGAEHVNNSYECD	-OH			
164	H-	AEHVNNSYECDIPIG	-OH			
165	H-	NNSYECDIPIGAGIC	-OH			
166	H-	ECDIPIGAGICASYQ	-OH			
167	H-	PIGAGICASYQTQTN	-OH			
168	H-	GICASYQTQTNSPRR	-OH			
169	H-	SYQTQTNSPRRARSV	-OH			**S2 (686-1273)**
170	H-	QTNSPRRARSVASQS	-OH			
171	H-	PRRARSVASQSIIAY	-OH			
172	H-	RSVASQSIIAYTMSL	-OH			
173	H-	SQSIIAYTMSLGAEN	-OH			
174	H-	IAYTMSLGAENSVAY	-OH			
175	H-	MSLGAENSVAYSNNS	-OH			
176	H-	AENSVAYSNNSIAIP	-OH			
177	H-	VAYSNNSIAIPTNFT	-OH			
178	H-	NNSIAIPTNFTISVT	-OH			
179	H-	AIPTNFTISVTTEIL	-OH			
180	H-	NFTISVTTEILPVSM	-OH			
181	H-	SVTTEILPVSMTKTS	-OH			
182	H-	EILPVSMTKTSVDCT	-OH			
183	H-	VSMTKTSVDCTMYIC	-OH			
184	H-	KTSVDCTMYICGDST	-OH			

185	H-	DCTMYICGDSTECSN	-OH			
186	H-	YICGDSTECSNLLLQ	-OH			
187	H-	DSTECSNLLLQYGSF	-OH			
188	H-	CSNLLLQYGSFCTQL	-OH			
189	H-	LLQYGSFCTQLNRAL	-OH			
190	H-	GSFCTQLNRALTGIA	-OH			
191	H-	TQLNRALTGIAVEQD	-OH			
192	H-	RALTGIAVEQDKNTQ	-OH			
193	H-	GIAVEQDKNTQEVFA	-OH	Pool 7	769-907	
194	H-	EQDKNTQEVFAQVKQ	-OH			
195	H-	NTQEVFAQVKQIYKT	-OH			
196	H-	VFAQVKQIYKTPPIK	-OH			
197	H-	VKQIYKTPPIKDFGG	-OH			
198	H-	YKTPPIKDFGGFNFS	-OH			
199	H-	PIKDFGGFNFSQILP	-OH			
200	H-	FGGFNFSQILPDPSK	-OH			
201	H-	NFSQILPDPSKPSKR	-OH			
202	H-	ILPDPSKPSKRSFIE	-OH			
203	H-	PSKPSKRSFIEDLLF	-OH			
204	H-	SKRSFIEDLLFNKVT	-OH			
205	H-	FIEDLLFNKVTLADA	-OH			
206	H-	LLFNKVTLADAGFIK	-OH			
207	H-	KVTLADAGFIKQYGD	-OH			
208	H-	ADAGFIKQYGDCLGD	-OH			
209	H-	FIKQYGDCLGDIAAR	-OH			
210	H-	YGDCLGDIAARDLIC	-OH			
211	H-	LGDIAARDLICAQKF	-OH			
212	H-	AARDLICAQKFNGLT	-OH			
213	H-	LICAQKFNGLTVLPP	-OH			
214	H-	QKFNGLTVLPPLLTD	-OH			
215	H-	GLTVLPPLLTDEMIA	-OH			
216	H-	LPPLLTDEMIAQYTS	-OH			
217	H-	LTDEMIAQYTSALLA	-OH			
218	H-	MIAQYTSALLAGTIT	-OH			
219	H-	YTSALLAGTITSGWT	-OH			
220	H-	LLAGTITSGWTFGAG	-OH			
221	H-	TITSGWTFGAGAALQ	-OH			
222	H-	GWTFGAGAALQIPFA	-OH			
223	H-	GAGAALQIPFAMQMA	-OH			
224	H-	ALQIPFAMQMAYRFN	-OH			
225	H-	PFAMQMAYRFNGIGV	-OH	Pool 8	899-1035	
226	H-	QMAYRFNGIGVTQNV	-OH			
227	H-	RFNGIGVTQNVLYEN	-OH			
228	H-	IGVTQNVLYENQKLI	-OH			
229	H-	QNVLYENQKLIANQF	-OH			
230	H-	YENQKLIANQFNSAI	-OH			
231	H-	KLIANQFNSAIGKIQ	-OH			

232	H-	NQFNSAIGKIQDSLS	-OH			
233	H-	SAIGKIQDSLSSTAS	-OH			
234	H-	KIQDSLSSTASALGK	-OH			
235	H-	SLSSTASALGKLQDV	-OH			
236	H-	TASALGKLQDVVNQN	-OH			
237	H-	LGKLQDVVNQNAQAL	-OH			
238	H-	QDVVNQNAQALNTLV	-OH			
239	H-	NQNAQALNTLVKQLS	-OH			
240	H-	QALNTLVKQLSSNFG	-OH			
241	H-	TLVKQLSSNFGAISS	-OH			
242	H-	QLSSNFGAISSVLND	-OH			
243	H-	NFGAISSVLNDILSR	-OH			
244	H-	ISSVLNDILSRLDKV	-OH			
245	H-	LNDILSRLDKVEAEV	-OH			
246	H-	LSRLDKVEAEVQIDR	-OH			
247	H-	DKVEAEVQIDRLITG	-OH			
248	H-	AEVQIDRLITGRLQS	-OH			
249	H-	IDRLITGRLQSLQTY	-OH			
250	H-	ITGRLQSLQTYVTQQ	-OH			
251	H-	LQSLQTYVTQQLIRA	-OH			
252	H-	QTYVTQQLIRAAEIR	-OH			
253	H-	TQQLIRAAEIRASAN	-OH			
254	H-	IRAAEIRASANLAAT	-OH			
255	H-	EIRASANLAATKMSE	-OH			
256	H-	SANLAATKMSECVLG	-OH			
257	H-	AATKMSECVLGQSKR	-OH	**Pool 9**	**1025-1163**	
258	H-	MSECVLGQSKRVDFC	-OH			
259	H-	VLGQSKRVDFCGKGY	-OH			
260	H-	SKRVDFCGKGYHLMS	-OH			
261	H-	DFCGKGYHLMSFPQS	-OH			
262	H-	KGYHLMSFPQSAPHG	-OH			
263	H-	LMSFPQSAPHGVVFL	-OH			
264	H-	PQSAPHGVVFLHVTY	-OH			
265	H-	PHGVVFLHVTYVPAQ	-OH			
266	H-	VFLHVTYVPAQEKNF	-OH			
267	H-	VTYVPAQEKNFTTAP	-OH			
268	H-	PAQEKNFTTAPAICH	-OH			
269	H-	KNFTTAPAICHDGKA	-OH			
270	H-	TAPAICHDGKAHFPR	-OH			
271	H-	ICHDGKAHFPREGVF	-OH			
272	H-	GKAHFPREGVFVSNG	-OH			
273	H-	FPREGVFVSNGTHWF	-OH			
274	H-	GVFVSNGTHWFVTQR	-OH			
275	H-	SNGTHWFVTQRNFYE	-OH			
276	H-	HWFVTQRNFYEPQII	-OH			
277	H-	TQRNFYEPQIITTDN	-OH			
278	H-	FYEPQIITTDNTFVS	-OH			

279	H-	QIITTDNTFVSGNCD	-OH			
280	H-	TDNTFVSGNCDVVIG	-OH			
281	H-	FVSGNCDVVIGIVNN	-OH			
282	H-	NCDVVIGIVNNTVYD	-OH			
283	H-	VIGIVNNTVYDPLQP	-OH			
284	H-	VNNTVYDPLQPELDS	-OH			
285	H-	VYDPLQPELDSFKEE	-OH			
286	H-	LQPELDSFKEELDKY	-OH			
287	H-	LDSFKEELDKYFKNH	-OH			
288	H-	KEELDKYFKNHTSPD	-OH			
289	H-	DKYFKNHTSPDVDLG	-OH			
290	H-	KNHTSPDVDLGDISG	-OH			
291	H-	SPDVDLGDISGINAS	-OH			
292	H-	DLGDISGINASVVNI	-OH			
293	H-	ISGINASVVNIQKEI	-OH			
294	H-	NASVVNIQKEIDRLN	-OH			
295	H-	VNIQKEIDRLNEVAK	-OH			
296	H-	KEIDRLNEVAKNLNE	-OH			
297	H-	RLNEVAKNLNESLID	-OH			
298	H-	VAKNLNESLIDLQEL	-OH			
299	H-	LNESLIDLQELGKYE	-OH			
300	H-	LIDLQELGKYEQYIK	-OH			
301	H-	QELGKYEQYIKWPWY	-OH			
302	H-	KYEQYIKWPWYIWLG	-OH	**Pool 10**	**1153-1273**	
303	H-	YIKWPWYIWLGFIAG	-OH			
304	H-	PWYIWLGFIAGLIAI	-OH			
305	H-	WLGFIAGLIAIVMVT	-OH			
306	H-	IAGLIAIVMVTIMLC	-OH			
307	H-	IAIVMVTIMLCCMTS	-OH			
308	H-	MVTIMLCCMTSCCSC	-OH			
309	H-	MLCCMTSCCSCLKGC	-OH			
310	H-	MTSCCSCLKGCCSCG	-OH			
311	H-	CSCLKGCCSCGSCCK	-OH			
312	H-	KGCCSCGSCCKFDED	-OH			
313	H-	SCGSCCKFDEDDSEP	-OH			
314	H-	CCKFDEDDSEPVLKG	-OH			
315	H-	DEDDSEPVLKGVKLH	-OH			
316	H-	DDSEPVLKGVKLHYT	-OH			
