# Supplementary material for: Comparison of rhesus and cynomolgus macaques as an infection model for COVID-19
Source: Nat Commun. 2021 Feb 24;12:1260. doi: 10.1038/s41467-021-21389-9 (PMC7904795; doi:10.1038/s41467-021-21389-9)
Supplement: Supplementary file 3 — Reporting Summary [file 41467_2021_21389_MOESM3_ESM.pdf]

nature research

Corresponding author(s): Miles Carroll

Last updated by author(s): 15/01/2021

Reporting Summary

Nature Research wishes to improve the reproducibility of the work that we publish. This form provides structure for consistency and transparency in reporting. For further information on Nature Research policies, see our Editorial Policies and the Editorial Policy Checklist.

Statistics

For all statistical analyses, confirm that the following items are present in the figure legend, table legend, main text, or Methods section.

n/a	Confirmed	
☐	☒	The exact sample size (*n*) for each experimental group/condition, given as a discrete number and unit of measurement
☐	☒	A statement on whether measurements were taken from distinct samples or whether the same sample was measured repeatedly
☐	☒	The statistical test(s) used AND whether they are one- or two-sided<br>*Only common tests should be described solely by name; describe more complex techniques in the Methods section.*
☒	☐	A description of all covariates tested
☒	☐	A description of any assumptions or corrections, such as tests of normality and adjustment for multiple comparisons
☐	☒	A full description of the statistical parameters including central tendency (e.g. means) or other basic estimates (e.g. regression coefficient) AND variation (e.g. standard deviation) or associated estimates of uncertainty (e.g. confidence intervals)
☐	☒	For null hypothesis testing, the test statistic (e.g. *F*, *t*, *r*) with confidence intervals, effect sizes, degrees of freedom and *P* value noted<br>*Give P values as exact values whenever suitable.*
☒	☐	For Bayesian analysis, information on the choice of priors and Markov chain Monte Carlo settings
☒	☐	For hierarchical and complex designs, identification of the appropriate level for tests and full reporting of outcomes
☒	☐	Estimates of effect sizes (e.g. Cohen's *d*, Pearson's *r*), indicating how they were calculated

Our web collection on statistics for biologists contains articles on many of the points above.

Software and code

Policy information about availability of computer code

Data collection	Nikon NIS-Ar package software (v4.30.01) was used to collect and analyse digital image data (ISH percentage of positively stained area).
Data analysis	GraphPad Prism 8.0, software package PESTLE v1.7, Hisat2 v2.10, featureCounts v2.0.0, Ensembl(release-94), EdgeR 3.6.3; FlowJo (version 9.7.6, BD Biosciences). OsiriX v12.0 was used to analyse CTscan images. ELISPOT plates were analysed using the CTL scanner and Immunospot®Analyzer software.

For manuscripts utilizing custom algorithms or software that are central to the research but not yet described in published literature, software must be made available to editors and reviewers. We strongly encourage code deposition in a community repository (e.g. GitHub). See the Nature Research guidelines for submitting code & software for further information.

Data

Policy information about availability of data

All manuscripts must include a data availability statement. This statement should provide the following information, where applicable:

- Accession codes, unique identifiers, or web links for publicly available datasets
- A list of figures that have associated raw data
- A description of any restrictions on data availability

All data and materials used in the analysis are presented in the main text and supplementary figures. Accession number PRJNA681111 is available at https://www.ncbi.nlm.nih.gov/bioproject/PRJNA681111

Field-specific reporting

Please select the one below that is the best fit for your research. If you are not sure, read the appropriate sections before making your selection.

- [x] Life sciences
- [ ] Behavioural & social sciences
- [ ] Ecological, evolutionary & environmental sciences

For a reference copy of the document with all sections, see nature.com/documents/nr-reporting-summary-flat.pdf

Life sciences study design

All studies must disclose on these points even when the disclosure is negative.

Sample size	No sample size calculation was performed. As this was an initial pilot experiment into SARS-CoV-2 in rhesus and cynomolgus macaques, 6 animals per species were selected. As per several other published early reports, minimal numbers of animals were used for sequential culls. We believe this provides important information regarding the rhesus and cynomolgus model of SARS-CoV-2 going forward.
Data exclusions	No data were excluded from the analysis
Replication	Animal experiments were not replicated due to ethical considerations when using animal modes. Samples for RT-qPCR and sgPCR were assayed in duplicate against a standard curve in triplicate. All replication attempts were successful.
Randomization	Animals were randomly allocated to groups according to social compatibility.
Blinding	Blinding was used in histopathological analysis. Numbers were randomly allocated to slides. Examination was carried out blinded and at random by independent pathologists. All the samples taken in life and at post-mortem were coded and blinded to the the investigators.

Reporting for specific materials, systems and methods

We require information from authors about some types of materials, experimental systems and methods used in many studies. Here, indicate whether each material, system or method listed is relevant to your study. If you are not sure if a list item applies to your research, read the appropriate section before selecting a response.

Materials & experimental systems

n/a	Involved in the study	
☐	☒	Antibodies
☐	☒	Eukaryotic cell lines
☒	☐	Palaeontology and archaeology
☐	☒	Animals and other organisms
☒	☐	Human research participants
☒	☐	Clinical data
☒	☐	Dual use research of concern

Methods

n/a	Involved in the study	
☒	☐	ChIP-seq
☐	☒	Flow cytometry
☒	☐	MRI-based neuroimaging

Antibodies

Antibodies used	Antibodies from BD Biosciences: CD28 (Cat 555725, Clone 28.2, Lot 9317139), CD49d (cat 555501, clone 9F10, lot 0086602), CD107a-AF488 (Cat 567007, clone H4A3, lot B214155), CD4 (Cat 552838, clone L200, lot 9171956), CD3-AF700 (Cat 557917, clone SP34-2, lot 9277122), TNFa-BUV395 (Cat 563996, clone MAb11, lot 8043611), CD56-BV605 (cat 742659, clone MY31, lot 0170610), HLA-DR-BUV395 (Cat 564040, clone C45-5, lot 0009310).<br>Antibodies from Beckman Coulter: CD159a-PC7 (cat B10246, clone Z199, lot 200051)<br>Antibodies from BioLegend: CD8-APCFire750 (cat 344746, clone SK1, lot B268052), CD69-BV510 (cat 310936, clone FN50, lot B266846), CD20-PeDazzle-594 (cat 302348, clone L27, lot B280782), GD-TCR-BV421 (Cat 331218, clone B1, lot B277884), IFNg-PeCy7 (cat 506518, clone B27, lot B278688), GM-CSF-PE (cat 502306, clone BVD2-21C11, lot B298254), IL-17-BV711 (cat 512328, clone BL168, lot B266567), CD11c-PE (Cat 301605, clone 3.9, lot B256068), CD14-APC (cat 301808, clone M5E2, lot B259538), CD16-BV786 (cat 302046, clone VNK80, lot B254002)<br>Antibodies from Miltenyi: IL-2-APC (cat 130-091-644, clone N7.48 A, lot 5200503406)
Validation	All monoclonal antibodies were titrated to determined optimal dilutions for the assay to achieve optimal signal to noise. Antibodies were used according to manufacturers instructions and validated by the manufacturer.<br><br>CD28<br>https://www.bdbiosciences.com/ds/pm/tds/555725.pdf<br>Manufacturer states cross-reactivity with cynomolgus and rhesus macaques

CD49d
https://www.bdbiosciences.com/ds/pm/tds/555501.pdf
Manufacturer states cross-reactivity with cynomolgus and rhesus macaques

IL-17
https://www.biolegend.com/en-us/global-elements/pdf-popup/brilliant-violet-711-anti-human-il-17a-antibody-7951?filename=Brilliant%20Violet%20711trade%20anti-human%20IL-17A%20Antibody.pdf&pdfgen=true
Previously used in primates by White et al 2021
http://dx.doi.org/10.1038/s41541-020-00262-8

GM-CSF
https://www.biolegend.com/en-us/global-elements/pdf-popup/pe-anti-human-gm-csf-antibody-916?filename=PE%20anti-human%20GM-CSF%20Antibody.pdf&pdfgen=true
Manufacturer states cross-reactivity with cynomolgus and rhesus macaques

CD69
https://www.biolegend.com/en-us/global-elements/pdf-popup/brilliant-violet-510-anti-human-cd69-antibody-8666?filename=Brilliant%20Violet%20510trade%20anti-human%20CD69%20Antibody.pdf&pdfgen=true
Manufacturer states cross-reactivity with cynomolgus and rhesus macaques

CD107a
Primate reactivity: http://www.ncbi.nlm.nih.gov/pubmed/20465573 (Magalhaes et al., 2010)

CD20
https://www.biolegend.com/en-us/global-elements/pdf-popup/pe-dazzle-594-anti-human-cd20-antibody-10436?filename=PEDazzletrade%20594%20anti-human%20CD20%20Antibody.pdf&pdfgen=true
Manufacturer states cross-reactivity with cynomolgus and rhesus macaques

GD TCR
https://www.biolegend.com/en-us/global-elements/pdf-popup/brilliant-violet-421-anti-human-tcr-gamma-delta-antibody-8705?filename=Brilliant%20Violet%20421trade%20anti-human%20TCR%20gammadelta%20Antibody.pdf&pdfgen=true
Manufacturer states cross-reactivity with cynomolgus and rhesus macaques

CD11c
https://www.biolegend.com/en-us/global-elements/pdf-popup/pe-anti-human-cd11c-antibody-563?filename=PE%20anti-human%20CD11c%20Antibody.pdf&pdfgen=true
Manufacturer states cross-reactivity with cynomolgus and rhesus macaques

CD14
https://www.biolegend.com/en-us/global-elements/pdf-popup/apc-anti-human-cd14-antibody-793?filename=APC%20anti-human%20CD14%20Antibody.pdf&pdfgen=true
Manufacturer states cross-reactivity with cynomolgus and rhesus macaques

CD16
https://www.biolegend.com/en-us/global-elements/pdf-popup/brilliant-violet-785-anti-human-cd16-antibody-7966?filename=Brilliant%20Violet%20785trade%20anti-human%20CD16%20Antibody.pdf&pdfgen=true
Manufacturer states cross-reactivity with cynomolgus and rhesus macaques
IL-2
https://www.miltenyibiotec.com/GB-en/products/il-2-antibody-anti-human-n7-48-a.html#apc:100-tests-in-1-ml
Manufacturer states cross-reactivity with NHPs

CD159a
https://bec-techdocs-prod.s3.us-west-2.amazonaws.com/techdoc/files/pi/B59648AB.pdf?AWSAccessKeyId=ASIA2KJI7HMZ7U7NYKFQ&Expires=1610552473&Signature=tdn66Yp%2FSr3i19AnSsgaCih5zwg%3D&x-amz-security-token=IQoJb3JpZ2luX2VjEBAaCXVzLXdlc3QtMiJHMEUCIQDIKo%2FNoWwc9QzLmuAxJaLiF4sG%2FUUBQaahJxI7esdINQIgG1gR6iW2tBvTZCGAcYKHq7tns0aN9BO3wCGnbDG%2BFI8q2wEI2P%2F%2F%2F%2F%2F%2F%2F%2F%2F%2FARACGgw3MDkyOTI0MDc2MDMiDHBst3Qfs80ydMcqbiqvASa4agD7naTUkq53ZYcTf92wVgWd%2BIzn0fmSj%2F662rgXsH5985Z89taTJxxFiCWEgnSWqL7DDSnBwWvSOZt4piSHqyTlPrpeL8Jua1Axql5awmQ4Yxj2cwF2r2j3vRxohpumv8E2rPgXQ8w8wKpkqxOceU5DsCNd5zZHaoY6ys43uDTb%2FB9I8IrMlNjM92xGiyElJLp8oZOhq%2FeN%2BiB1%2BiuprxCaYxMCdJUiweqBcSMw7J78%2FwU64AEsZBDJ7YBk4ebsl3ZDHod9GBeym8PTvVdnPZ2qAiz3UBTyULVFoWRn6JNb%2Ftscayfrq3lZvN1o677PwBfdIRNwsNesJpjKASeztJvmSNpBz4SrwlHvowWKzn7qOIYwg%2BBaXJAI%2Bf7xX5XFQKM9JIURNf2mjs%2Bak%2Fpbheltq3W%2FV44OliFdISqgKWSPnw7EGHK3m%2Bpdc09jaUUmBwTAmj%2Ftnt3BEoM1cAsYO8m%2FkSyqA7FjONvIkkw0Retit5cWr6hn7mlIMlGRpy8tHdEtjt0uVxZv8%2BHKtbLac%2BZAkYWycQ%3D%3D
Previously used in macaques by Choi et al 2008
https://www.ncbi.nlm.nih.gov/pmc/articles/PMC2566626/

HLA-DR
https://www.bdbiosciences.com/ds/pm/tds/564040.pdf
Manufacturer states cross-reactivity with cynomolgus and rhesus macaques

CD56<br>https://www.bdbiosciences.com/eu/tds/742659<br>Previously used in macaques by Autissier et al 2010<br>http://www.pubmedcentral.nih.gov/articlerender.fcgi?artid=2930593&tool=pmcentrez&rendertype=abstract<br><br>TNFa<br>https://www.bdbiosciences.com/ds/pm/tds/563996.pdf<br>Manufacturer states cross-reactivity with cynomolgus and rhesus macaques<br><br>IFNg<br>https://www.biolegend.com/en-us/global-elements/pdf-popup/pe-cyanine7-anti-human-ifn-gamma-antibody-5938?filename=PECyanine7%20anti-human%20IFN-gamma%20Antibody.pdf&pdfgen=true<br>Manufacturer states cross-reactivity with cynomolgus and rhesus macaques<br><br>CD3<br>https://www.bdbiosciences.com/ds/pm/tds/557917.pdf<br>Manufacturer states cross-reactivity with cynomolgus and rhesus macaques<br><br>CD8<br>https://www.biolegend.com/en-us/global-elements/pdf-popup/apc-fire-750-anti-human-cd8-antibody-13035?filename=APCFiretrade%20750%20anti-human%20CD8%20Antibody.pdf&pdfgen=true<br>Manufacturer states cross-reactivity with cynomolgus and rhesus macaques<br><br>CD4<br>https://www.bdbiosciences.com/ds/pm/tds/552838.pdf<br>Manufacturer states cross-reactivity with cynomolgus and rhesus macaques

Eukaryotic cell lines

Policy information about cell lines

Cell line source(s)	Vero/hSLAM cells [ECACC 04091501], Vero/E6 cells [ECACC 85020206]
Authentication	Cell lines were obtained from the European Collection of Authenticated Cell Cultures (ECACC) PHE, Porton Down, UK.<br>All stock sourced from ECACC has been manufactured according to established SOPs and quality control released following a set of established tests according ISO 17025, including<br>- Testing for bacteria and fungi<br>- Testing for mycoplasma<br>- Authentication testing: STR DNA profiling for human cell lines and DNA barcoding for non-human and human cell lines to confirm species.
Mycoplasma contamination	All cell lines used were confirmed negative for mycoplasm.
Commonly misidentified lines<br>(See ICLAC register)	No commonly misidentified cells lines were used in this study.

Animals and other organisms

Policy information about studies involving animals; ARRIVE guidelines recommended for reporting animal research

Laboratory animals	Cynomolgus macaques (Macaca fascicularis), male and female; Rhesus macaques (macaca mulatta), male and female. 2-4 years of age.
Wild animals	No wild animals were used during this study.
Field-collected samples	No field-collected samples were used during this study.
Ethics oversight	All experimental work was conducted under the authority of a UK Home Office approved project licence (PDC57C033) that had been subject to local ethical review at PHE Porton Down by the Animal Welfare and Ethical Review Body (AWERB) as required by the Home Office Animals (Scientific Procedures) Act 1986.<br><br>The study protocol was approved by ethical review at PHE Porton Down by the Animal Welfare and Ethical Review Body (AWERB).

Note that full information on the approval of the study protocol must also be provided in the manuscript.

Flow Cytometry

Plots

Confirm that:

- [x] The axis labels state the marker and fluorochrome used (e.g. CD4-FITC).
- [x] The axis scales are clearly visible. Include numbers along axes only for bottom left plot of group (a 'group' is an analysis of identical markers).
- [x] All plots are contour plots with outliers or pseudocolor plots.
- [x] A numerical value for number of cells or percentage (with statistics) is provided.

Methodology

Sample preparation	PBMCs were isolated from whole blood anticoagulated with heparin (132 Units per 8 ml blood) (BD Biosciences, Oxford, UK) using standard methods. Of note is that the material used for density gradient centrifugation was adjusted dependent on the macaque species, with a Ficoll Histopaque gradient (GE Healthcare, USA) used with Rhesus macaque blood and a Percoll gradient (GE Healthcare) used with cynomolgus macaques. Mononuclear cells (MNC) were isolated from spleen and lung tissue samples using an OctoMACS tissue dissociation device (Miltenyi Biotec). Lung tissue samples were dissected into approximately 5mm3 pieces and incubated for one hour in a solution of 772.8 U/ml collagenase + 426 U/ml DNase (both from Sigma) diluted in Earle's balanced salt solution supplemented with 200 mg/ml Calcium Chloride (Gibco, Life Technologies, Renfrew, UK), at 37ºC with continual gentle mixing of the tube. The homogenised solution was passed through a 70 µm cell filter (BD Biosciences) and the mononuclear cells separated by Ficoll Histopaque density gradient centrifugation. PBMCs and MNC isolated from tissues were stored at −180 °C until resuscitated for analysis.
Instrument	BD LSRII Fortessa
Software	FlowJo V9.7.6, PESTLE V1.7, GraphPad Prism V8.01
Cell population abundance	A minimum of 100,000 lymphocytes were collected in the FSC/SSC gate for analysis.
Gating strategy	Cytokine producing T-cells were identified using a forward scatter-height (FSC-H) versus side scatter-area (SSC-A) dot plot to identify the lymphocyte population, to which appropriate gating strategies were applied to exclude doublet events, non-viable cells and B cells (CD20+). For ICS analysis, sequential gating through CD3+, followed by CD4+ or CD8+ gates were used before individual cytokine gates to identify IFN-γ, IL-2, TNF-α, GM-CSF and IL-17, CD107a and CD69 stained populations. In immunophenotyping data sets, classical-, non-classical-monocytes and monocyte derived dendritic cells (mDCs) were identified by FSC and SSCcharacteristics and by the expression pattern 808 of HLA-DR, CD14, CD16 and CD11c within the live CD3-, CD20- population. Similarly, natural killer cells subsets were identified by expression of CD8, CD159a, CD56 and CD16 within live CD3- lymphocyte subsets. Polyfunctional cells were identified using Boolean gating combinations of individual cytokine-producing CD4 or CD8 T-cells.

- [x] Tick this box to confirm that a figure exemplifying the gating strategy is provided in the Supplementary Information.
